# Pramipexole-Induced Hypothermia Reduces Early Brain Injury via PI3K/AKT/GSK3β pathway in Subarachnoid Hemorrhage rats

**DOI:** 10.1038/srep23817

**Published:** 2016-03-30

**Authors:** Junwei Ma, Zhong Wang, Chenglin Liu, Haitao Shen, Zhouqing Chen, Jia Yin, Gang Zuo, Xiaochun Duan, Haiying Li, Gang Chen

**Affiliations:** 1 Department of Neurosurgery & Brain and Nerve Research Laboratory, The First Affiliated Hospital of Soochow University, 188 Shizi Street, Suzhou 215006, China; 2 Department of Neurosurgery, Suzhou Kowloon Hospital Affiliated Shanghai Jiao Tong University, Suzhou, 266021, China

## Abstract

Previous studies have shown neuroprotective effects of hypothermia. However, its effects on subarachnoid hemorrhage (SAH)-induced early brain injury (EBI) remain unclear. In this study, a SAH rat model was employed to study the effects and mechanisms of pramipexole-induced hypothermia on EBI after SAH. Dose-response experiments were performed to select the appropriate pramipexole concentration and frequency of administration for induction of mild hypothermia (33–36 °C). Western blot, neurobehavioral evaluation, Terminal deoxynucleotidyl transferase-mediated dUTP nick end labeling (TUNEL) and Fluoro-Jade B (FJB) staining were used to detect the effects of pramipexole-induced hypothermia on SAH-induced EBI, as well as to study whether controlled rewarming could attenuate these effects. Inhibitors targeting the PI3K/AKT/GSK3β pathway were administered to determine whether the neuroprotective effect of pramipexole-induced hypothermia was mediated by PI3K/AKT/GSK3β signaling pathway. The results showed that intraperitoneal injection of pramipexole at 0.25 mg/kg body weight once per 8 hours was found to successfully and safely maintain rats at mild hypothermia. Pramipexole-induced hypothermia ameliorated SAH-induced brain cell death, blood-brain barrier damage and neurobehavioral deficits in a PI3K/AKT/GSK3β signaling-dependent manner. Therefore, we may conclude that pramipexole-induced hypothermia could effectively inhibit EBI after SAH in rats *via* PI3K/AKT/GSK3β signaling pathway.

Subarachnoid hemorrhage (SAH), a serious threat to human life and health, is an acute hemorrhagic cerebrovascular disease due to rupture of intracranial vessels caused by a variety of factors^1^,^2^. Currently, with the continuous improvement of surgical techniques and medical devices, the recovery rate for SAH from aneurysm ruptures is steadily rising, but the mortality and morbidity of SAH are still surprisingly high^3^. Recent studies have shown that early brain injury (EBI) is the main cause of morbidity and mortality in SAH patients within 24 to 72 hours^4^,^5^. A growing body of evidence has shown that apoptosis contributed to the progression of EBI after SAH^6^,^7^. However, to date, effective strategies to prevent brain cells from these apoptosis-promoting mechanisms are lacking.

For centuries, hypothermia has been considered to be a valuable clinical treatment^8^. Depending on the temperature, hypothermia can be divided into mild hypothermia (33–36 °C), moderate hypothermia (28–32 °C), severe hypothermia (<28 °C)^9^. Experimental studies in recent years have suggested that mild hypothermia has a brain-protective effect^10^,^11^,^12^,^13^. However, in clinical practice there few beneficial effects have been realized^14^. Hence, the optimization of applications of existing drug-induced hypothermia or develop/screening new drugs for inducing hypothermia may provide an effective tool for clinical treatment. In addition, current hypothermia research focuses on cerebral ischemia and traumatic brain injury, but whether hypothermia, specifically under SAH conditions, plays a neuroprotective effect is still unclear^15^,^16^.

Drugs commonly used for inducing therapeutic hypothermia include cannabinoid, opioid receptor agonists, transient receptor potential vanilloid, neurotensin, hormone agonists, dopamine receptor agonists, gas that induces hypothermia, and adenosine and adenine nucleotides^17^. Among dopamine receptor agonists, both talipexole and pramipexole has been shown as antiparkinsonian drugs and confer neuroprotection in several experimental paradigms, but the responsible mechanisms remain unknown^18^,^19^. In addition, previous studies have shown that talipexole could inhibit brain damage due to ischemia through inducing hypothermia^20^. However, besides as an agonist selective for dopamine receptor D2, talipexole also acts as α2-adrenoceptor agonist and 5-HT3 antagonist^21^, which may need to be considered as non-negligible side effects and limitations, while pramipexole has high selectivity for interacting with dopamine D2 subfamily receptors and has little interaction with adrenergic or serotonergic receptors^22^. Furthermore, pramipexole have been implicated in causing hypothermia in free-fed rats^23^. Thus, pramipexole may be neuroprotective by direct effects or indirect effects related to its hypothermic effects.

In the case of cardiac ischemia-reperfusion, sub-low body temperature at 34 °C can effectively suppress myocardial injury caused by ischemia-reperfusion through activation of PI3K signaling pathway^24^. In addition, hydroxysafflor yellow A and tetramethylpyazine analogues regulate Bcl-2/Bax levels by activating PI3K/AKT/GSK3β signaling pathway to inhibit caspase-dependent apoptosis pathway in brain cells, and thereby inhibit apoptosis induced by ischemia and reperfusion^25^,^26^. Furthermore, pramipexole pretreatment could increase Bcl-2 and inhibit caspase-3-dependent apoptosis in human neuroblastoma SH-SY5Y cells treated with 1-methyl–4-phenylpyridinium^19^. However, whether pramipexole induced-hypothermia could inhibit caspase3-dependent apoptosis *via* PI3K/AKT/GSK3β signaling pathway, and thus exert a neuroprotective effect has not been reported.

Therefore, we sought to test whether pramipexole could induce hypothermia and the effects of pramipexole on EBI in a rat SAH model in this study.

## Results

### Dose Response

**Figure 1:**
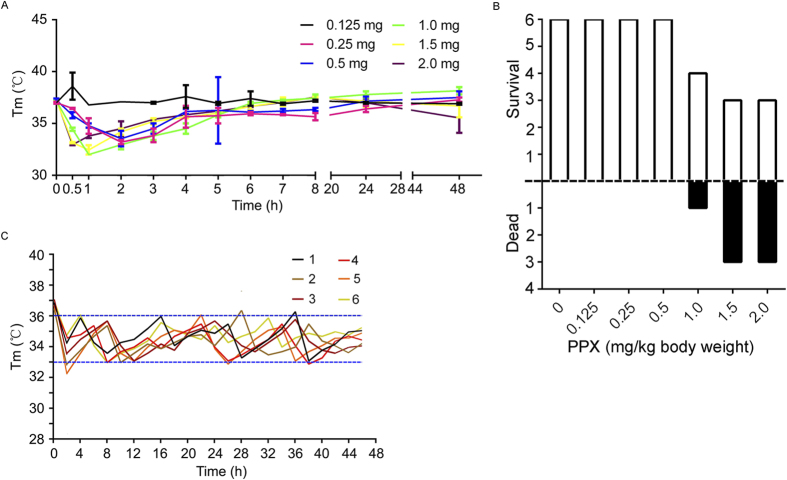
Pramipexole-induced hypothermia and its effects on brain cell apoptosis. (**A**) Rats separately received intraperitoneal injection of pramipexole at 0, 0.125, 0.25, 0.5, 1.0, 1.5 and 2.0mg/kg body weight once 8 hours, and the body temperature was continuously monitored for 48 hours. Data are expressed as means ± SEM. (**B**) The mortality of each group shown in (**A**). A total of six rats each group. Among them, the survival ones were marker as blank, while dead as black. (**C**) Body temperature monitoring of SAH rats receiving 0.25 mg/kg body weight pramipexole once 8 hours.

Administration of pramipexole at a dose range of 0.25 to 2.0 mg/kg body weight resulted in mild to moderate hypothermia (Fig. 1A). The mortality of each group was shown in Fig. 1B. Then, we chose the dose of 0.25 mg/kg body weight in the following study because it could lead to hypothermia safely. In addition, SAH rats also could maintain a mild hypothermia (33–36 °C) after receiving 0.25 mg/kg body weight of pramipexole once 8 hours (Fig. 1C). The data showed that 0.25 mg/kg body weight pramipexole could safely and effectively induce hypothermia in SAH rats.

### Protective effects of pramipexole on SAH-induced EBI in rats

**Figure 2:**
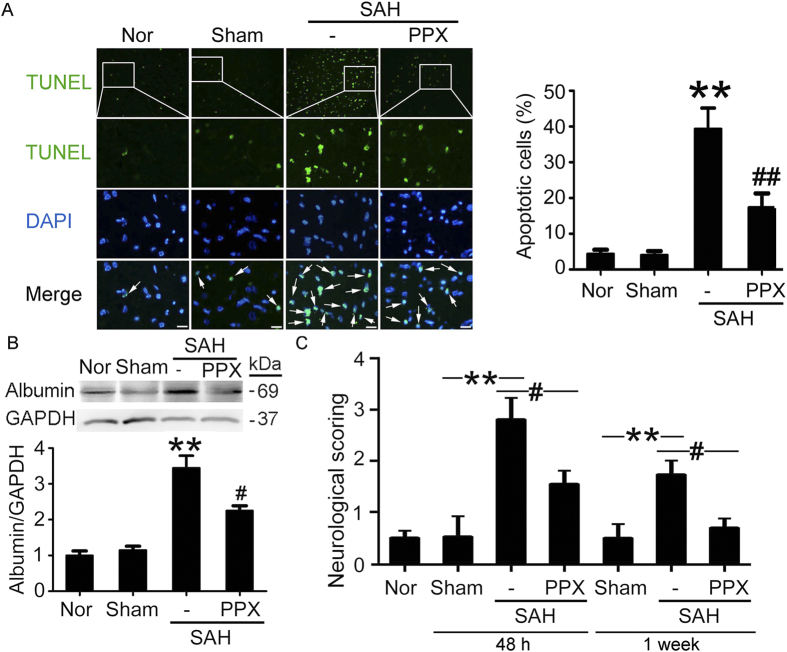
Effects of pramipexole-induced hypothermia and brain cell apoptosis. (**A**) TUNEL staining. Arrows point to TUNEL-positive cells, Scale bar = 32 μm. Percentage of TUNEL-positive cells was shown. Data are expressed as means ± SEM. **p < 0.01 *vs*. sham group; ^##^*p* < 0.01 *vs*. SAH group, n = 6. PPX: pramipexole. (**B**) Western blot detection of albumin in brain tissues. Statistical analysis of albumin in brain tissues was shown. Data are expressed as means ± SEM. **p < 0.01 *vs*. sham group; ^#^*p* < 0.05 *vs*. SAH group, n = 6. (**C**) Neurobehavioral evaluation of each group at 48 h and 1 week after SAH model established. Data are expressed as means ± SEM. **p < 0.01 *vs*. sham group; ^#^*p* < 0.05 *vs*. SAH group, n = 6.

Terminal deoxynucleotidyl transferase-mediated dUTP nick end labeling (TUNEL) staining showed that, compared with sham group, increased apoptotic index was detected in SAH group. Remarkably, treatment with pramipexole induced a significant decrease in TUNEL-positive cells in the brain compared with SAH group (Fig. 2A). In addition, western blot analysis and neurological assessment showed that pramipexole could effectively suppress SAH-induced albumin extravasation and neurobehavioral dysfunction (Fig. 2B,C). In summary, pramipexole could effectively suppress brain cell death and ameliorate the blood-brain barrier damage and neurobehavioral dysfunction caused by SAH.

### Rewarming abolished the protective effects of pramipexole on SAH-induced EBI in rats

**Figure 3:**
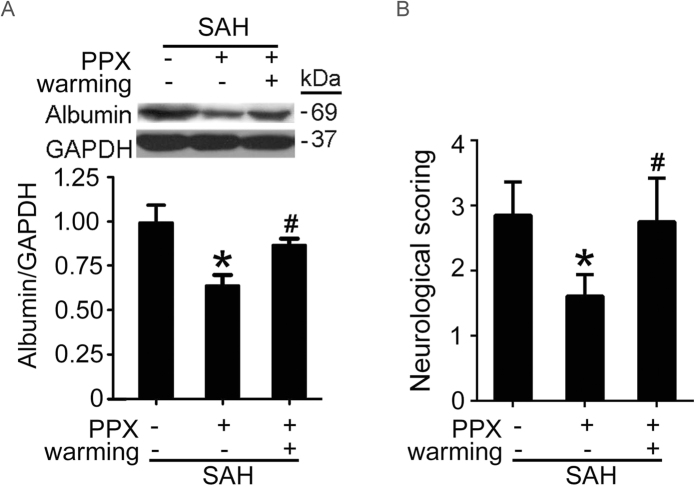
Effects of pramipexole-induced hypothermia and controlled rewarming on albumin leakage through the blood-brain barrier and neurobehavioral deficits after SAH. Western blot detection of albumin in brain tissues (**A**) and neurobehavioral evaluation (**B**) were performed. In (**A,B**), Data are presented as means ± SEM. *p < 0.05 *vs*. SAH group; ^#^*p* < 0.05 *vs*. SAH + PPX group, n = 6. PPX: pramipexole.

In six of the pramipexole-injected rats, the temperature was actively controlled so that hypothermia was prevented with temperatures kept at values similar to those of SAH rats for 48 hours. Western blot analysis and neurological assessment showed that, in rewarming group, pramipexole could not effectively suppress albumin extravasation and neurobehavioral dysfunction induced by SAH (Fig. 3A,B), suggesting that the protective effects of pramipexole were mediated by its production of hypothermia, at least partially.

### Pramipexole-induced hypothermia may exert anti-apoptosis effects *via* PI3K/AKT/GSK3β signaling pathway in SAH rats

**Figure 4:**
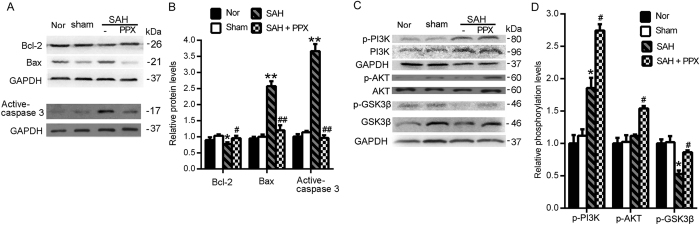
Western blot analysis of pramipexole-induced hypothermia on the apoptosis-related proteins and PI3K/AKT/GSK3β pathway in the brains of SAH rats. (**A**) Western blot analysis of the protein levels of Bcl-2, Bax and active-caspase-3 in brain tissues. (**B**) Statistical analysis of Bcl-2, Bax and caspase-3 activation in brain tissues. Data are expressed as means ± SEM. *p < 0.05, **p < 0.01 *vs*. sham group; ^#^*p* < 0.05, ^##^*p* < 0.01 *vs*. SAH group, n = 6. (**C**) Western blot detection of phosphorylation of PI3K, AKT and GSK3β in brain tissues. (**D**) Statistical analysis of phosphorylation of PI3K, AKT and GSK3β in brain tissues. Phosphorylation levels were evaluated by the ratio of phosphoprotein to total protein. Data are expressed as means ± SEM. *p < 0.05 *vs*. sham group; ^#^*p* < 0.05 *vs*. SAH group, n = 6. PPX: pramipexole.

**Figure 5:**
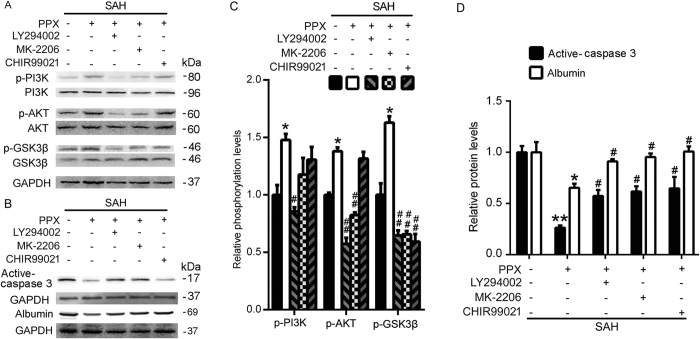
In presence of pramipexole-induced hypothermia, effects of PI3K/AKT/GSK3β inhibitors on caspase-3 activation and albumin leakage in the brains of SAH rats. (**A**) Western blot detection of LY294002, MK-2206 and CHIR99021 blockage of phosphorylation of PI3K, AKT and GSK3β. (**C**) Statistical analysis of PI3K, AKT and GSK3β phosphorylation in brain tissues. Data are expressed as means ± SEM. *p < 0.05 *vs*. SAH group; #*p* < 0.05, ^##^*p* < 0.01 *vs*. SAH + PPX group; n = 6. (**B**) Western blot detection of active caspase-3 and albumin in brain tissues. (**D**) Statistical analysis of activation of caspase-3 and albumin leakage in the brain tissues. Data are expressed as means ± SEM. *p < 0.05, **p < 0.01 *vs*. SAH group; ^#^*p* < 0.05 *vs*. SAH + PPX group; n = 6. PPX: pramipexole.

Western blot analysis showed that, compared with the sham group, the Bcl-2 protein levels were significantly reduced in the SAH group, while Bax protein levels and caspase-3 activation increased significantly in the SAH group. Compared with the SAH group, pramipexole-induced hypothermia could effectively prevent the SAH-induced inhibition of Bcl-2 and increases of Bax and caspase-3 activation (Fig. 4A,B). In addition, western blot results also showed that, compared with the sham group, the SAH group demonstrated increased PI3K phosphorylation, no obvious change of the phosphorylation of AKT, significantly reduced GSK3β phosphorylation. Compared with the SAH group, SAH + pramipexole treatment group demonstrated significantly elevated levels of phospho-PI3K, -AKT, and -GSK3β (Fig. 4C,D). Furthermore, to clear the possible mechanisms of pramipexole-induced anti-apoptotic effects, we applied LY294002, MK-2206 and CHIR99021 to block PI3K, AKT and GSK3β activation, respectively. The effects of the various blocking agents were detected by western blot. The results showed that, LY294002 could effectively suppress phosphorylation of PI3K, AKT and GSK3β; MK-2206 could effectively suppress phosphorylation of AKT and GSK3β; and CHIR99021 could effectively inhibit the phosphorylation of GSK3β in the brains of SAH rats (Fig. 5A,C). In addition, in the presence of any of these blockers, pramipexole-induced hypothermia could not effectively suppress caspase-3 activation and blood-brain barrier damage induced by SAH (Fig. 5B,D). In summary, pramipexole-induced hypothermia may inhibit caspase-dependent apoptosis *via* PI3K/AKT/GSK3β signaling pathways in the brains of SAH rats.

### Blocking PI3K/AKT/GSK3β pathway interrupted the protective effects of pramipexole-induced hypothermia on SAH-induced EBI in rats

**Figure 6:**
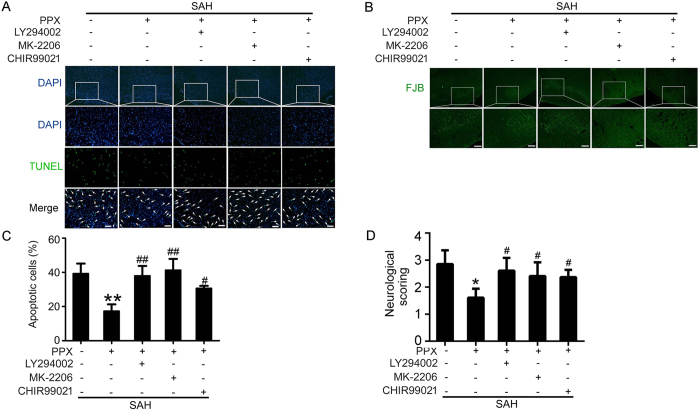
In presence of pramipexole-induced hypothermia, effects of PI3K/AKT/GSK3β inhibitors on brain cell death and neurobehavioral deficits after SAH. (**A,C**) TUNEL staining. Arrows point to TUNEL-positive cells. Scale bar = 32 μm. Percentage of TUNEL-positive cells was shown. Data are expressed as means ± SEM. **p < 0.01 *vs*. SAH group; ^#^*p* < 0.05, ^##^*p* < 0.01 *vs*. SAH + PPX group, n = 6. (**B**) FJB staining. Scale bar = 32 μm. (**D**) Neurobehavioral score of each group. Data are expressed as means ± SEM. *p < 0.01 vs. SAH group; ^#^*p* < 0.05 vs. SAH + PPX group, n = 6.

TUNEL and Fluoro-Jade B (FJB) staining showed that in presence of any blocker of PI3K/AKT/GSK3β pathway, pramipexole-induced hypothermia could not effectively suppress SAH-induced brain cell apoptosis and necrosis, and neurobehavioral dysfunction (Fig. 6A–D).

## Discussion

**Figure 7:**
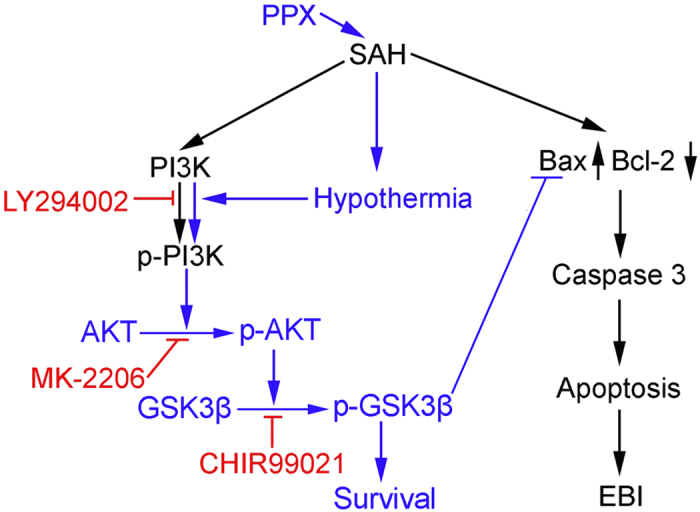
Schematic diagram of the pramipexole-induced hypothermia-mediated neuroprotective mechanisms in SAH rats. Blue colored letters and arrows indicate the pramipexole-induced hypothermia-mediated anti-apoptotic effect, and black colored letters and arrows indicate the SAH-induced pro-apoptotic pathway. To some extent, PI3K was activated under SAH conditions, but PI3K/AKT/GSK3β pathway is incomplete, and pramipexole-induced hypothermia promotes PI3K/AKT/GSK3β pathway activation in SAH. Red colored letters and arrows indicate the effects of PI3K inhibitor LY294002, AKT inhibitor MK-2206, and GSK3β inhibitor CHIR99021. PPX: pramipexole.

The present study demonstrated that the dopamine receptor agonist pramipexole, when administered after SAH, induced mild hypothermia effectively and safely. It decreased SAH-induced brain cell death via PI3K/AKT/GSK3β pathway, and then reduced EBI. In addition, PI3K was phosphorylated to some extent under SAH, suggesting PI3K activation maybe a self-help measure in brain cells after SAH, which is enhanced by pramipexole-induced hypothermia. We reasoned that pramipexole could be regarded as a potential drug candidate for mild hypothermia induction that would like contribute to a beneficial outcome of SAH patients. To the best of our knowledge, this is the first study demonstrating such involvement (Fig. 7).

It has been reported that 30 of 51 in neurologic intensive care patients showed an elevation of the body temperature (>37.9 °C) within 24 hours after termination of the cooling study by an intravascular cooling device^27^, suggesting that there may be rewarming injury during cooling treatment. Also, as shown in Fig. 1C, there were obvious temperature fluctuations during pramipexole-induced hypothermia. However, the body temperature always maintained between 33–36 °C, which was the dominant temperature range of mild hypothermia. In addition, it was reported that there was temporal patterns of body temperatures in the acute stage of stroke^28^, and stroke severity determines body temperature in acute stroke response^29^, suggesting an existing temperature fluctuations after stroke. These reports may explain the temperature fluctuations during pramipexole-induced hypothermia. And our results suggested that there was not rewarming injury during the intervals of each injection of pramipexole.

As we all known, the human brain is an organ with high metabolic demands and high heat production, and brain metabolism is very sensitive to temperature fluctuations^30^. Low body temperature slows down the metabolism rates of glucose, proteins and lipids. Experimental studies have shown that as the temperature drops by 1 °C, cerebral metabolic rate drops 6–7%, and thus lowers the brain oxygen consumption, and finally exerts a neuroprotective effect in the acute phase of brain injury^31^. Commonly applied methods for hypothermia are physical, intravascular and drug-induced. Physical cooling is through the use of an ice pack or ice blanket or alcohol to reduce the temperature on the skin surface. This method is simple, but cooling the skin surface is poorly adaptive, unbearable in patients, the cooling effect is slow, an improper control of the temperature, and often causes skeletal muscle tumor^32^. Intravascular cooling is through the means of intravenous equipment with cold liquid or temperature sensors within the catheter through the blood stream. It features fast cooling, precise control of temperature changes, but is also invasive, results in easy bleeding and prone to infection, which, in particular, is not available for patients taking anticoagulants^33^. Drug-induced hypothermia refers to the use of drugs to lower body temperature 2–6 °C below normal. Currently drug-induced hypothermia has been studied using relevant animal experimental models in multiple neuronal fields, such as cerebral ischemia, cerebral hemorrhage, epilepsy, and neurodegenerative diseases^34^.

Previous studies also showed that drug-induced hypothermia has some side effects, such as ventricular tachycardia, ventricular fibrillation, suppressed immune function, infection, electrolyte imbalance, glucose metabolism and skeletal muscle tumor. In a rat cerebral ischemia model, adenosine 5′-monophosphate (AMP)-induced hypothermia was incapable of suppressing brain damage caused by ischemia, but increased infarct size instead, and increased rat mortality^35^. Similarly, adenosine 5′-triphosphate (ATP) could induce mild hypothermia, but exacerbate brain injury caused by ischemia^36^. In addition, cannabinoid receptor agonists may cause drowsiness, lethargy, decreased movement, limb rigidity and movement dysfunction. Opioid receptor agonists may cause vasodilation leading to lower blood pressure, arrhythmia. Vanilloid receptor agonists have little effect on blood pressure, but the hypothermia effect is slow, requires frequent dosing, yet excessive administration leads to neurotoxic effects. Hence, screening new drugs for inducing hypothermia is extremely urgent. Dopamine receptor agonists, talipexole and pramipexole have been shown as antiparkinsonian drugs and confer neuroprotection in several experimental paradigms^18^,^19^. Current studies have shown that talipexole can inhibit brain damage caused by ischemia through inducing hypothermia^20^. In this study, we explored the effects of pramipexole on brain damage after SAH for the first time. However, the dose-response experiment suggested that overdose of pramipexole led to some rats died. At the same time, we found that over-dose of pramipexole (1.0–2.0 mg/kg body) did not induced lower body temperature, compared with 0.25 mg/kg body pramipexole, suggesting that over-dose of pramipexole may kill rats via its effect as a dopamine receptor agonist. Based on the dose-response experiment, we found that 0.25 mg/kg body pramipexole safely and effectively induced hypothermia and attenuated brain injury after experimental SAH in rats. However, the risk of using drugs like pramipexole in SAH-related patients needs further evaluation.

Studies have shown that in SAH rats, PI3K signaling pathway is activated, and thus inhibits neuronal apoptosis, indicating that PI3K activation may be a self-protective mechanism of neurons in SAH^37^. Consistent with these reports, we found that phosphorylation of PI3K was increased in SAH. Meanwhile, GSK3β, a downstream substrate of PI3K, declined significantly. Pramipexole-induced hypothermia could effectively increase phosphor-PI3K, -AKT, -GSK3β levels, and playing a protective role in the brain. In summary, our study showed that PI3K is activated to some extent, but PI3K/AKT/GSK3β pathway is incomplete. In addition, as shown in Fig. 4C,D, pramipexole appeared to recover PI3K/AKT/GSK3β pathway and SAH-induced apoptosis. These results may be interpreted in two ways: PPX is directly recovering PI3K-Akt signaling, or is recovering brain cell viability that is reflected (secondarily) by the recovery of PI3K-Akt pathway. To further distinguish the mechanism of pramipexole action, we administered PI3K/AKT/GSK3β pathway inhibitors, which blocked PI3K/AKT/GSK3β pathway and interrupted the protective effects of pramipexole-induced hypothermia on SAH-induced EBI as shown in the present study. Based on these results, we concluded that pramipexole-induced hypothermia could effectively inhibit EBI after SAH in rats via PI3K/AKT/GSK3β signaling pathway.

As lacking of correlative reports of BBB permeability to the three inhibitors (LY294002, MK-2206 and CHIR99021) used in this study, the amount of the inhibitors reaching brain and whether it reach an inhibitory concentration in the brain once plagued us. To prove the BBB permeability to the three inhibitors, we have treated both sham-rats and SAH-rats with the three inhibitors via intraperitoneal injection at the same dosage of that used in this study. The results showed little blocking effect of the three inhibitors in sham-rats, but significant blocking effects in SAH group (data not shown). Excitingly, as shown in Fig. 5A,C, there were significant blocking effects of the three inhibitors on their corresponding putative target kinases, suggesting that these inhibitors entered the brain parenchyma and reach an inhibitory concentration in the brain under SAH condition. It may be explained by the report that micro-disruptions of the BBB could allow increased access of some drugs to the immediate environment around those disruptions^38^. As we all know, the BBB acts as a selective permeability interface between the central nervous system (CNS) and circulating blood. BBB disruption itself contributes to the onset and progression of SAH-induced EBI^39^. In this sense, the BBB itself should be considered as a therapeutic target. Meanwhile, disruptions of the BBB could also actually facilitate making drug cross the BBB into the brain parenchyma^38^. So a better understanding of how BBB permeability differ in SAH patients would allow appropriate adjustments in drug dosages and fewer CNS side effects.

In the present study, the temperature maintained between 33–36 °C, which was slightly higher than 32–34 °C recommended by the American Heart Association guidelines for therapy hypothermia^40^. However, it was also proved that therapy hypothermia with the target temperature of 34–35 °C, the dominant temperature range in the present study (Fig. 1C), was more easily attainable, feasible, safe, and efficient in patients^41^.

This study has several limitations. First, to avoid additional brain injury, noninvasive rectal temperature was adopted in this experiment instead of brain temperature. It is difficult to indicate the effect of pramipexole on brain temperature in the current study. Second, this experiment was performed on healthy rats without comorbidities common in people susceptible to a brain hemorrhage. Third, the aim of the study was to investigate whether pramipexole could induce therapeutic hypothermia pharmacologically. The safety and efficacy of this method therefore were not compared with the conventional physical cooling techniques. Fourth, based on the current experimental design, it is difficult to exclude the direct neuroprotective effect of pramipexole independent of hypothermia. Last, as shown in Figs 2B and 3A, hypothermia protected SAH-induced BBB disruption, suggesting that hypothermia may exert the protection via endothelial cells. However, the effect of hypothermia on endothelial cells and the potential mechanisms, especially whether hypothermia protects endothelial cells also via PI3K/AKT/GSK3β signaling, need further investigation.

Therapeutic mild hypothermia could be induced pharmacologically by pramipexole. Pramipexole-induced hypothermia inhibited brain cell death via activation of the PI3K/AKT/GSK3β pathway, and then attenuated SAH-induced EBI. Our findings therefore may provide a further option for effective induction of therapeutic hypothermia after SAH by pharmacologic means alone or in combination with physical cooling.

## Methods

### Animals

One hundred and twenty-six adult male Sprague-Dawley (SD) rats weighing between 300–350 g were purchased from the Animal Center of Chinese Academy of Sciences, Shanghai, China. The animal experimental protocols were approved by the Animal Care and Use Committee of Soochow University, and complied with the Guide for the Care and Use of Laboratory Animals by the National Institutes of Health, including all use, care and operative procedures. The rats were housed in temperature- and humidity-controlled animal quarters with a 12-h light/dark cycle. Every effort was made to minimize the number of animals used and their suffering.

### SAH model

**Figure 8:**
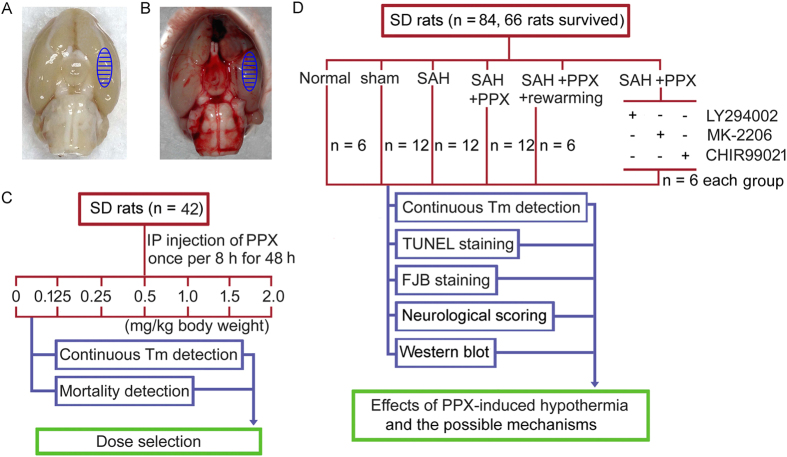
Experimental Design. (**A,B**) Schematic representation of the areas taken for assay. (**A**) Sham group. (**B**) Subarachnoid hemorrhage (SAH) group. (**C**) Design of dose-response experiment. (**D**) Experimental design for studying the effects of pramipexole-induced hypothermia and the possible mechanisms. Briefly, sixty-six rats (84 rats were used, 66 rats were survived) were randomly divided into 4 groups: normal group (n = 6), sham group (n = 12), SAH group (n = 12), and SAH + pramipexole group (n = 36). Among the 36 rats in SAH + pramipexole group, 24 rats were randomly subdivided into 4 groups: SAH + pramipexole + rewarming group (n = 6), SAH + pramipexole + LY294002 group (n = 6), SAH + pramipexole + MK-2206 group (n = 6), and SAH + pramipexole + CHIR99021 group (n = 6). PPX: pramipexole.

An autologous blood SAH model was performed as previously reported^42^. Firstly, rat heads were fastened in a stereotactic frame with the mouthpiece at 0° after anesthesia by an intraperitoneal injection of chloral hydrate (36 mg/100 g body weight). By using an automatic heating pad, the body temperature of the animals was maintained at 37.5 ± 0.5 °C. Femoral artery was cannulated to gauge mean arterial blood pressure and to gain blood samples. The experimental SAH model was produced using stereotaxic insertion of a needle with a rounded tip and a side hole into the prechiasmatic cistern. The needle was inserted 7.5 mm anterior to the bregma in the midline at an angle of 45° in the sagittal plane with the needle hole facing the right side. Penetration continued until the tip reached the base of the skull, 2–3 mm anterior to the chiasma and retracted 0.5 mm. Loss of cerebrospinal fluid (CSF) and bleeding from the midline vessels could be prevented by plugging the burr hole with bone wax before inserting the needle if needed. The impaired femoral artery was sutured carefully using a 12–0 proline under the operating microscope to avoid ligaturing the impaired femoral artery. Then, a total of 0.3 ml of non-heparinized fresh autologous arterial blood from femoral artery was injected evenly into the prechiasmatic cistern over 20 s using a syringe pump under aseptic conditions while 0.3 ml saline were injected in the same manner for sham group. The animals were allowed to recover for 45 min after the operation. After the operation, 5 ml of 0.9% NaCl was injected subcutaneously to prevent dehydration. It was observed in this experiment that inferior basal temporal lobe was always stained by blood. Therefore, the brain tissue adjacent to or under the blood clots was taken for the analysis in our study. Schematic representation of the areas taken for assay was shown in Fig. 8A,B. Animals underwent assessment of EBI at 48 hours after SAH.

### Experiment design

#### Part I. Dose-response experiment 42 SD rats were used for dose-response experiment

These rats were randomly divided into seven groups with six rats per group. Each group separately received pramipexole at a dose of 0, 0.125, 0.25, 0.5, 1.0, 1.5 and 2.0 mg/kg body weight by intraperitoneal injection once per 8 hours for 48 hours. And, the body temperature and the mortality of each group were checked every one hour for selection of the appropriate drug concentration (Fig. 8C).

#### Part II Effects of pramipexole-induced hypothermia

We injected rats intraperitoneally with pramipexole (0.25 mg/kg body weight) immediately after SAH and once per 8 hours for 48 hours. To minimize the number of animals used and because of that pramipexole was dissolved in normal saline, vehicle or placebo treatment was not contained in this study. Body temperature was continuously monitored to verify whether pramipexole also could induce hypothermia under SAH conditions. At 48 hours after SAH, brain cell apoptosis and necrosis were studied by TUNEL and FJB staining, blood brain barrier integrity was studied by western blot analysis of albumin leakage in brain tissues, and neurobehavioral evaluation was also performed to study the effects of pramipexole-induced hypothermia on SAH induced-EBI. To test a prolonged neuroprotective effect of pramipexole-induced hypothermia, neurobehavioral evaluation was performed on the other six rats in sham group, SAH group and SAH + pramipexole at one week after SAH. In addition, the protein levels of Bcl-2 and Bax, the activation of caspase-3, and the phosphorylation of PI3K/AKT/GSK3β in brain tissues were also detected by western blot to examine the possible mechanisms of the action of pramipexole-induced hypothermia (Fig. 8D).

#### Part III. Effects of rewarming

To further check whether the effects of pramipexole was mediated by its induced hypothermia, rats were rewarmed with a heating lamp to the same temperatures observed in the SAH group. Assessment of EBI was performed at 48 hours after SAH, as mentioned above (Fig. 8D).

#### Part IV. Effects of PI3K/AKT/GSK3β pathway inhibitors

To examine the mechanism of action of pramipexole, we administered PI3K inhibitor LY294002, AKT inhibitor MK-2206 and GSK3β inhibitor CHIR99021 simultaneously with the first intraperitoneal injection of pramipexole or vehicle immediately after SAH. Rats underwent EBI assessment at 48 hours after SAH, as mentioned above (Fig. 8D).

### Drug treatment

Pramipexole (Boehringer Ingelheim, CA, U.S.), prepared in normal saline at a concentration of 12.5% stock solution, was injected intraperitoneally at a dose range of 0, 0.125, 0.25, 0.5, 1.0, 1.5 and 2.0 mg/kg body weight. PI3K inhibitor LY294002 (Selleck, S1105, TX, U.S.) was prepared in DMSO at a concentration of 36% stock solution and injected intraperitoneally at a dose of 100 mg/kg body weight. AKT inhibitor MK-2206 (Selleck, S1078, TX, U.S.) was prepared in captisol at a concentration of 30% stock solution and injected intraperitoneally at 120 mg/kg body weight. GSK3β inhibitor CHIR99021 (Selleck, S2924, TX, U.S.) was prepared in citrate-buffered saline at a concentration of 15% stock solution and injected intraperitoneally at a dose of 48 mg/kg body weight. The mixture of equal volumes of DMSO, captisol and citrate-buffered saline was used as vehicle, which did not effect of the phosphorylation of PI3K, AKT, and GSK3β (data not shown).

### TUNEL staining

Cell apoptosis in rat brain tissue was detected by TUNEL staining according to the manufacturer’s protocol (DeadEnd Flurometric kit, Promega, WI, U.S.). Briefly, brain tissues were paraffin embedded and sectioned, and then heated and dewaxed. After dewaxed, the sections were washed 3 times with PBS, and then incubated with TUNEL-staining at 37 °C for 60 min. Nuclei were stained with DAPI (Southern Biotech, Birmingham, AL, U.S.) mounting medium after washed 3 times with PBST (5 min per wash) at room temperature. Finnally, the sections were visualized by a fluorescence microscope (OLYMPUS BX50/BX-FLA/DP70; Olympus Co., Japan.) and TUNEL-positive cells were counted by an observer who was blind to the experimental groups. To evaluate the extent of cell apoptosis, 6 microscopic fields per sample were examined and photographed in parallel for TUNEL-positive cell counting, and the apoptotic index was defined as the average number of TUNEL-positive cells in each section.

### FJB staining

FJB staining (Histo-Chem Inc., Jefferson, AR, U.S.) staining served as a marker of neuronal necrosis. Brain sections were deparaffinized and rehydrated. After incubation with deionized water for 1 min, the slides were incubated in 0.06% K permanganate for 15 min. Slides were then rinsed in deionized water and immersed in fluoro-jade working solution (0.1% acetic acid) for 30 min. Then they were washed and dried in an incubator (50–60 °C) for 10 min. Sections were cleared in xylene and coverslipped with a non-aqueous, low-fluorescence, styrene-based mounting medium (DPX, Sigma-Aldrich, MO, U.S.). Microscopy of the stained tissue sections was performed by an experienced pathologist blind to the experimental condition.

### Neurobehavioral evaluation

At 48 hours and 1 week after SAH, all the rats in experiments were examined for behavioral impairment using a scoring system and monitored for appetite, activity, and neurological defects, as previously reported^42^. For details, please see Table 1.

### Western blot analysis

Western blot analysis was performed as described previously^43^.Briefly, the brain samples were mechanically lysed in RIPA lysis buffer for western blot (Beyotime Institute of Biotechnology, Shanghai, China). The protein concentrations were measured by the bicinchoninic acid (BCA) method using enhanced BCA protein assay kit (Beyotime Institute of Biotechnology, Shanghai, China). Protein samples (50 μg/lane) were loaded on a 12% SDS-polyacrylamide gel, separated, and electrophoretically transferred to a polyvinylidene difluoride (PVDF) membrane (Millipore Corporation, Billerica, MA, U.S.), which was blocked with 5% bovine serum albumin (BSA, BIOSHARP, Hefei, China) for 1 hour at room temperature. The membrane was then incubated overnight at 4 °C with primary antibodies. The primary antibodies against Bcl-2 (Abcam, ab18210, Cambridge, U.K.), Bax (Abcam, ab32503, Cambridge, U.K.), active-caspase3 (Abcam, ab2302, Cambridge, U.K.), albumin (Abcam, ab106582, Cambridge, U.K.), PI3K (Santa Cruz, sc-7189, Shanghai, China), Phos-PI3K (CST, 4228, MA, U.S.), AKT (Abcam, ab32505, Cambridge, U.K.), Phos-AKT (Abcam, Ab4060, Cambridge, U.K.), GSK3β (CST, 9832, MA, U.S.), Phos-GSK3β (CST, 5558, MA, U.S.) were diluted 1:2000. The primary antibody against GAPDH was diluted 1:5000 and served as loading controls. The membrane was then incubated with an HRP-conjugated secondary antibody (diluted 1:5,000, Santa Cruz Biotechnology, Shanghai, China.) for 2 hours at room temperature. The band signal was developed using enhanced chemiluminescence (ECL) kit (Beyotime, Shanghai, China). The relative quantity of proteins was analyzed using Image J and normalized to that of loading controls. Phosphorylation levels were evaluated by the ratio of phosphoprotein to total protein.

### Statistical analysis

Values are presented as means ± SEM. SPSS 11.5 (SPSS Inc., Chicago, IL, U.S.) was used for statistical analysis. The Mann-Whitney U test was used to compare behavior scores among groups. Statistical comparisons between groups were performed using one-way analysis of variance followed by either a Dunnett’s or a Tukey’s post hoc test, the former for comparisons to a single control group, the latter to compare across multiple groups. A probability of P < 0.05 was considered statistically significant.

## Additional Information

**How to cite this article**: Ma, J. *et al*. Pramipexole-Induced Hypothermia Reduces Early Brain Injury via PI3K/AKT/GSK3β pathway in Subarachnoid Hemorrhage rats. *Sci. Rep.* **6**, 23817; doi: 10.1038/srep23817 (2016).

## Figures and Tables

**Table 1:** Behavior and activity scores.

Category	Behavior	Score
Appetite	Finished meal	0
	Left meal unfinished	1
	Scarcely ate	2
Activity	Walk and reach at least three corners of the cage	0
	Walk with some stimulations	1
	Almost always lying down	2
Deficits	No deficits	0
	Unstable walk	1
	Impossible to walk	2
